# Introduction to Photonics: Principles and the Most Recent Applications of Microstructures

**DOI:** 10.3390/mi9090452

**Published:** 2018-09-11

**Authors:** Iraj Sadegh Amiri, Saaidal Razalli Bin Azzuhri, Muhammad Arif Jalil, Haryana Mohd Hairi, Jalil Ali, Montree Bunruangses, Preecha Yupapin

**Affiliations:** 1 Division of Materials Science and Engineering, Boston University, Boston, MA 02215, USA; amiri@bu.edu; 2 Department of Computer System & Technology, Faculty of Computer Science & Information Technology, University of Malaya, 50603 Kuala Lumpur, Malaysia; saaidal@um.edu.my; 3 Department of Physics, Faculty of Science, Universiti Teknologi Malaysia, 81300 Johor Bahru, Malaysia; arifjalil@utm.my; 4 Faculty of Applied Sciences, Universiti Teknologi Mara, Pasir Gudang Campus, 81750 Johor, Malaysia; haryanahairi@gmail.com; 5 Laser Centre, IBNU SINA ISIR, Universiti Teknologi Malaysia, 81310 Johor Bahru, Malaysia; djxxx_1@yahoo.com; 6 Faculty of Industrial Education, Rajamangala University of Technology Phranakorn, Bangkok 10300, Thailand; montree.b@rmutp.ac.th; 7 Computational Optics Research Group, Advanced Institute of Materials Science, Ton Duc Thang University, District 7, Ho Chi Minh City, Vietnam; 8 Faculty of Electrical & Electronics Engineering, Ton Duc Thang University, District 7, Ho Chi Minh City, Vietnam

**Keywords:** light, photon, communications, waveguides, fibers, biosensors

## Abstract

Light has found applications in data transmission, such as optical fibers and waveguides and in optoelectronics. It consists of a series of electromagnetic waves, with particle behavior. Photonics involves the proper use of light as a tool for the benefit of humans. It is derived from the root word “photon”, which connotes the tiniest entity of light analogous to an electron in electricity. Photonics have a broad range of scientific and technological applications that are practically limitless and include medical diagnostics, organic synthesis, communications, as well as fusion energy. This will enhance the quality of life in many areas such as communications and information technology, advanced manufacturing, defense, health, medicine, and energy. The signal transmission methods used in wireless photonic systems are digital baseband and RoF (Radio-over-Fiber) optical communication. Microwave photonics is considered to be one of the emerging research fields. The mid infrared (mid-IR) spectroscopy offers a principal means for biological structure analysis as well as nonintrusive measurements. There is a lower loss in the propagations involving waveguides. Waveguides have simple structures and are cost-efficient in comparison with optical fibers. These are important components due to their compactness, low profile, and many advantages over conventional metallic waveguides. Among the waveguides, optofluidic waveguides have been found to provide a very powerful foundation for building optofluidic sensors. These can be used to fabricate the biosensors based on fluorescence. In an optical fiber, the evanescent field excitation is employed to sense the environmental refractive index changes. Optical fibers as waveguides can be used as sensors to measure strain, temperature, pressure, displacements, vibrations, and other quantities by modifying a fiber. For some application areas, however, fiber-optic sensors are increasingly recognized as a technology with very interesting possibilities. In this review, we present the most common and recent applications of the optical fiber-based sensors. These kinds of sensors can be fabricated by a modification of the waveguide structures to enhance the evanescent field; therefore, direct interactions of the measurand with electromagnetic waves can be performed. In this research, the most recent applications of photonics components are studied and discussed.

## 1. Introduction

**Figure 1:**
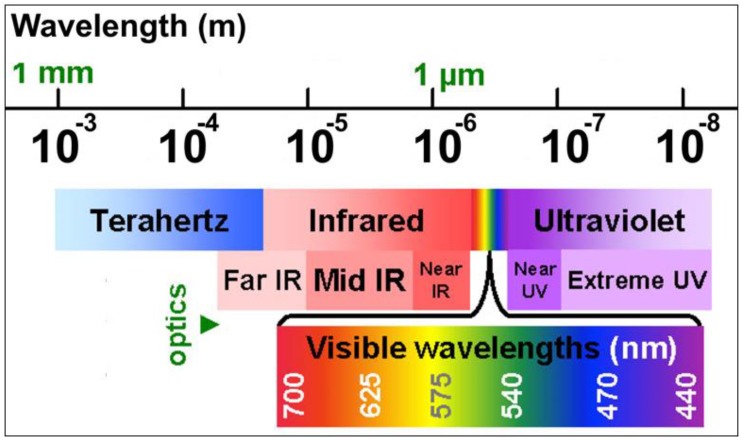
The electromagnetic spectrum.

The role of light is significant in our lives today. The importance of light cannot be taken for granted because it is vital to most aspects of our contemporary society. It is used everywhere whether it be building, telecommunication, transportation, entertainment, or clothing. Light has applications in data transmission, such as optical fibers and in optoelectronics. It is used in compact disc players where a laser reflecting off of a CD transforms the returning signal into music. It is also used in laser printing and digital photography. Connections between computers and telephone lines are possible with the help of light (fiber-optic cables). It is used in optical fiber lasers, optical fiber interferometers, optical fiber modulators, and sensors. Light is used in the medical field for image production used in hospitals and in lasers that are used for optometric surgery [1]. Light consists of a series of electromagnetic waves, with particle behavior under certain circumstances. Light is the range of wavelengths in the electromagnetic spectrum (Figure 1).

Photonics is essentially the science that involves generation of a photon (light), its detection, as well as manipulation via transmission, emission, signal processing, modulation, switching, amplification, and sensing. Most importantly, photonics involves the proper use of light as a tool for the benefit of humans [2,3]. Most photonics applications, even though they cover all technical applications over the entire electromagnetic spectrum, range from near-infrared light to visible region. The term “photonics” was derived from the root word “photon”, which connotes the tiniest entity of light analogous to an electron in electricity. Just as the electronics revolutionized the 20th century, photonics is doing the same in the 21st century. Photonics is made up of many different technologies including optical fibers, lasers, detectors, quantum electronics, fibers, and materials [4]. 

The term photonics was first used to designate a field of research area responsible for utilizing light to perform tasks that are conventionally related to the traditional sphere of electronics, like telecommunications, information processing, and so on. Studies in the field of potonics began in 1960 after the discovery of lasers. Other progress followed including optical fibers for transmitting information, the laser diode in the 1970s, as well as erbium fiber amplifiers. These developments made the foundation for the industrial revolution in the telecommunications sector during the late 20th century and supplied the internet infrastructure. Although created before the 1980s, the word photonics was used commonly for the first time in the 1980s as network operators of telecommunications embraced fiber-optic data transmission. Photonics came into being when the “IEEE Lasers and Electro-Optics Society” came up with a journal called “Photonics Technology Letters” towards the end of the 18th century. Through the years, until 2001 with the dot-com crash, research was primarily focused on optical fiber telecommunication. Nevertheless, the field of photonics has a broad range of scientific and technological applications. These include chemical and biological sensors, laser manufacturing, medical therapy and medical diagnosis, optical computing and displaying technology. Advancement of photonics is possible due to the current success recorded concerning the development of silicon photonics. Photonics is related to opto-mechanics, electro-optics, quantum electronics and quantum optics. Nevertheless, these fields mean different things to both the scientific as well as the business community. Quantum optics is often concerned with fundamental theoretical research areas. Photonics, on the other hand, deals with applied research and progress. Optoelectronics is used to refer to the circuits or devices consisting of both electrical and optical components. The word “electro-optics” was utilized in the past to specifically relate to nonlinear interactions between electrical and optical devices. These devices include bulk crystal modulators and later include advanced imaging sensors that are typically employed by both government and private individuals in surveillance activities [5,6].

Photonics is said to be an “All-Pervasive” technology because it allows unlimited light to travel faster than the electrons that are used in electronic computer chips, which means that optical computers will compute thousands of times faster than any electronic computers because of the physical limitations of electronic conduction. More wavelengths can be packed into an optical fiber to allow an increase in the transmission bandwidth that can be in conventional copper wires. There is no electromagnetic interference in light compared to electrons in copper wires [7,8].

## 2. Applications of Photonics

Photonics have uses in almost every aspect of our life, ranging from daily life to highly innovative science. For instance, information processing, telecommunications, light detection, metrology, lighting, spectroscopy, photonic computing, holography, medical field (surgery, vision correction, health monitoring and endoscopy), fighting machinery, visual art, agriculture, laser material processing, robotics, and biophotonics. Similar to the way electronics have been used extensively since the creation of earlier transistors of 1948, the exceptional use of photonics continuously increases. Economically significant uses of photonic devices include fiber optic telecommunications, optical data storage, displays, optical pumping of high-power lasers and laser printing. Prospective applications of photonics are practically limitless and include medical diagnostics, organic synthesis, information, and communication, as well as fusion energy [9,10]:

- Telecommunication: optical down-converter to microwave, and optical fiber communications.
- Medical applications: laser surgery, poor eyesight correction, tattoo removal and surgical endoscopy.
- Manufacturing processes in industries: involves the use of laser in welding, cutting, drilling, and many surface modification techniques.
- Building and construction: smart structures, laser range finding, and laser leveling.
- Space exploration and aviation: including astronomical telescopes.
- Military operations: command and control, IR sensors, navigation, mine laying, hunt and salvage, and discovery.
- Metrology: range finding, frequency and time measurements.
- Photonic computing: printed circuit boards, and quantum computing.
- Micro-photonics and nanophotonics.

These typically include solid-state devices and photonic crystals [11]. In simple terms, photonics is currently solving and addressing the challenges of a modern world. Photonics enhances the quality of life; it safeguards our health, security, and safety, it drives our economic growth, and it creates jobs as well as global effectiveness. Photonics technology enhances the quality of life in many areas. Specific areas are communications and information technology, advanced manufacturing, defense, health and medicine, and energy [12,13]. Photodetectors are used to detect light. They can be very slow, as in the case of solar cells that are used in harvesting sunlight energy, or very fast like photodiodes that are very fast and are employed in communications in conjunction with digital cameras. Numerous others centered on quantum, thermal, photoelectric and chemical areas also exist. Photonics likewise involves research on photonic systems. The term photonics system has found its application in optical communication systems [14]. 

## 3. Advances in Photonics

**Figure 2:**
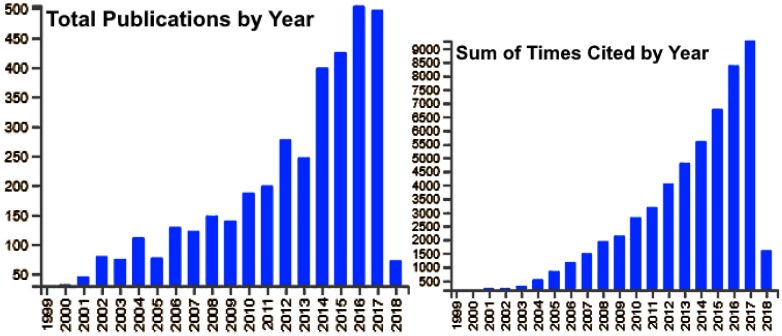
Publications and citation trends in Photonics (Source: Thomson Reuters Web of Science).

There has been an exponential growth in the research activities in the field of photonics and optics over the years, as illustrated by the publication and citation trends from the Thomson Reuters web of science database (Figure 2). 

Photonic networks are the backbone of data dissemination, specifically in the modern and upcoming wireless communication systems. Photonic networks continue to gain interest for distribution of data from, say, central location to a remote antenna unit at base stations. While the demand for wireless photonic systems continues to rise, there is a need for implementation of low-cost systems [15]. Two of the most popular data transmission methods in wireless photonic systems are digital baseband and RoF (Radio-over-Fiber) optical communication. In addition, further emerging fields are opto-atomics, in which there is an integration of both atomic and photonic devices. Opto-atomics applications include precise time-keeping. Opto-mechanics, metrology, and navigation, as well as polaritonics, are different from photonics due to the presence of polarization as the primary carrier of information. Microwave photonics is considered to be an emerging research field. Microwave photonics is an enabling technology for the generation, control, distribution, measurement, and detection of microwave signals. It also deals with the operation of new systems and devices [16,17,18,19]. Part of the various functionalities facilitated by photonics, microwave measurements centered on photonics can offer greater performance regarding broad frequency coverage, significant direct bandwidth, high immunity to electromagnetic interference (EMI) and low frequency-dependent loss. Photonic microwave measurements therefore have been widely investigated in recent times. Moreover, several new methodologies have been offered to address the challenges confronting electronic solutions [20]. Plasmon lasers are among the categories of optical frequency amplifiers that send strong, penetrating, and guiding superficial plasmons underneath the diffraction walls. The interactions between light energy and matter can be intensely improved by the tightly held electric fields in plasmon lasers. This will also bring substantial innovative possibilities to data storage, bio-sensing, optical communications and photolithography [21]. Because they can generate high-intensity nano-scale electromagnetic radiation in a fraction of a second, the modern development of plasmon lasers today has sparked the investigation of nanoscience and technology. This would enable more feature sizes than the conventional lasers [22,23]. They could also be used to package additional information onto storage media such as hard disks or DVDs [24,25]. The mid-IR spectroscopy offers a principal means for biological structure analysis as well as nonintrusive measurements. For instance, the broad cross-section for absorption allows for the detection of traces of vapors at the order of parts-per-trillion (ppt) as well as parts per- billion (ppb). 

## 4. Structure, Types, and Applications of Optical Fibers

Optical fibers are flexible filaments made of very clear glass and can carry information in the form of light from one point to another. They are hair-thin structures formed through the formation of preforms, which are glass rods made into fine threads of glass and secured by plastic coatings. Various vapor deposition processes are employed by fiber manufacturers to draw the preform. The thread drawn from this preform is then usually wrapped into a cable configuration, which is then placed into an operative situation for years of dependable performance [26]. 

**Figure 3:**
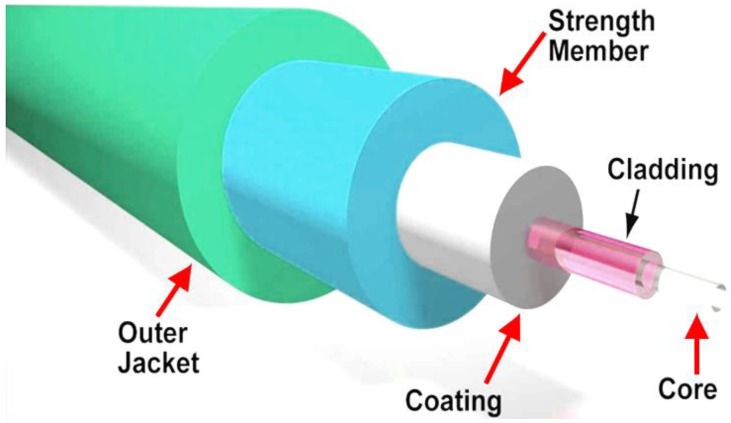
Structure of optical fiber.

The two most important components of optical fibers are the core and the cladding. The “core”, which is the axial part of the optical fiber, is made up of silica glass. The optical fiber core is that area of the fiber where light is transmitted. Sometimes, doping elements are used to modify the fiber refractive index, thereby changing the light velocity through the fiber. The “cladding”, on the other hand, is the layer that surrounds the core completely. The cladding refractive index is less than that of the core. This enables the light inside the core to strike the core-cladding interface at a “bouncing angle”, is confined inside the core by the total internal reflection, and keeps moving in the appropriate direction along the fiber length to a certain point. The cladding is usually surrounded by another layer known as “coating,” which normally is comprised of protective polymer films coated during the process of fiber drawing, before being in contact with any surface. Additional protective layers of “Buffers” are further applied on top of the polymer coatings as shown in Figure 3 [27].

The mechanism of the modifications on the fiber surface can be characterized through the transmission spectrum measurement of the fibers. There are so many different possible configurations of fibers corresponding to different application purposes. The most important classification considers fibers as either single-mode fibers and multimode fibers. The concept of application-specific fibers was invented at Bell Laboratories in the mid-1990s, and this is followed by an introduction of fibers designed for network applications. These next designs that are used mainly for signal transmission in communications consist of 10-Gbps laser-optimized multimode fibers (OM3), Zero Water Peak Fiber (ZWPF), Non-Zero Dispersion Fibers (NZDF), and fibers that are specially designed for the marine application. Specially designed fibers, like erbium-doped fibers, and dispersion compensating fibers perform tasks that supplement the transmission fibers. The differences between the different transmission fibers are responsible for variations in the number and range of different wavelengths or pathways via which the light is received or transmitted; this is the distance at which a signal can travel without being amplified or regenerated, and the speed at which this signal can travel. 

The silica fibers are the common type of fibers that can transmit light with wavelengths below the mid-infrared range [28]. The silica as an optical waveguide is a strongly absorbing material for wavelengths above 2 µm [29]. This is due to multiphoton absorption that causes vibrational resonance; however, there are different glass materials that can be used to fabricate the optical fibers in which these materials can transmit light at a longer wavelength [30]. The crystalline materials and hollow fiber waveguides are good candidates to perform these kinds of transmissions [31]. For instance, glasses such as the chalcogenides, which may have different compositions of sulfides, selenides or tellurides, have substantially lower vibration frequencies and therefore lower photon energy compared to silica [32]. This is due to the higher mass of chalcogenide ions compared to oxygen ions. Examples of these materials can be such as arsenic (As) or germanium (Ge), where the infrared absorption of the materials starts at longer wavelengths. Hollow waveguide fibers, however, can be used for single-mode transmission, although fibers transmitting light of wavelengths larger than 2 µm can be manufactured using either glass or crystalline materials [33]. The mid-infrared optical fibers have disadvantages of high fabrication cost, less mechanical robustness, and higher propagation loss in the optical communication wavelength range at 1.5 µm compared to silica fibers [34]. These are available as bare fibers and fiber patch cable and are presenting additional protection and fiber connectors at the end of their length. These fibers are mostly multimode waveguides and can be used for particular applications; however, there are many challenges in the fabrication of mid-infrared optical fibers in single-mode construction [35]. Recently, scientists are facing many technical challenges with fabricating the kind of fibers with air holes. For instance, omniguide fibers [36], hollow IR transmitting fibers [37] and holely fibers [38] can provide additional functionalities that are not available in other conventional fibers such as solid core fibers. These have unusual guiding structures and can support new light propagation features applicable to novel photonic devices such as lasers and transmitters. Infrared fibers such as Chalcogenide (CIR) [39] and Polycrystalline (PIR) [40] can be made of two different core materials. In CIR fibers, a high transmittance can be achieved in the wavelength range between 2 to 6 µm, where these fibers exhibit very low optical loss and high flexibility. The PIR fibers show a high transmittance in the range between 4 to 18 µm. In these two types of fibers, the light leakage is eliminated by implementing a special design of the core and cladding, which allows for a high damage tolerance to withstand damages from other even more intense sources such as continuous-wave CO_2_ lasers. Infrared fibers have many applications in imaging devices, thermal imaging, evanescent wave sensors and chemical species analyzers [41,42].

**Figure 4:**
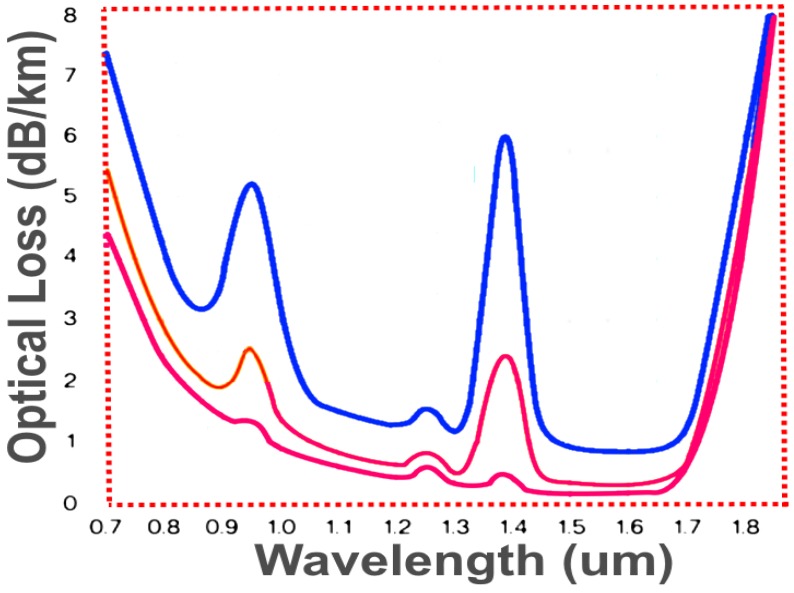
Attenuation against wavelength transmission windows.

Some critical parameters affect the performance of optical fibers transmission systems. These parameters and their specifications vary by fiber type and depend upon the intended use. Two of the more significant parameters of fibers are fiber dispersion and attenuation. Attenuation is the decrease in optical power when it propagates from one place to another. High attenuations affect the distance at which signals can be transmitted. Figure 4 shows the variation in attenuation with wavelengths for a wide range of fiber optic cables. 

Dispersion, on the other hand, is inversely related to the bandwidth and refers to the fiber to carry information. Single-mode fibers are associated with a chromatic dispersion that causes pulse spreading due to the various colors of light passing through the fiber at different speeds. Similarly, multimode fibers are related to the modal dispersion that causes pulse spreading due to the geometry of a multimode fiber core, which allows for the multiple modes lasers to simultaneously separate and propagate at the fiber interface. 

Multimode fibers are the first fibers to be produced on a commercial scale. They are called multimode fibers just because they allow several modes or rays of light to propagate through the waveguide simultaneously. These types of fibers have a much wider core diameter, when compared to the single-mode fibers, and allow for the higher number of modes. Multimode fibers are easier to couple than single-mode fibers. Multimode fibers can be classified into graded-index and step-index fibers. Graded-index multimode fibers make use of the differences in compositions of the glass inside the fiber core and recompense the different path lengths of the modes. They offer more bandwidth than step index fibers. Step-index multimode fibers were the first cords designed but are too slow regarding most applications because of the dispersion caused by the different path lengths of the various modes. Step-index fibers are barely used in modern telecommunications. Multimode fibers that are employed in communications possess the core size of 50 or 62.5 microns. The big core sizes allow the fibers to support many diagonal electromagnetic modes for a given polarization and frequency. 

**Figure 5:**
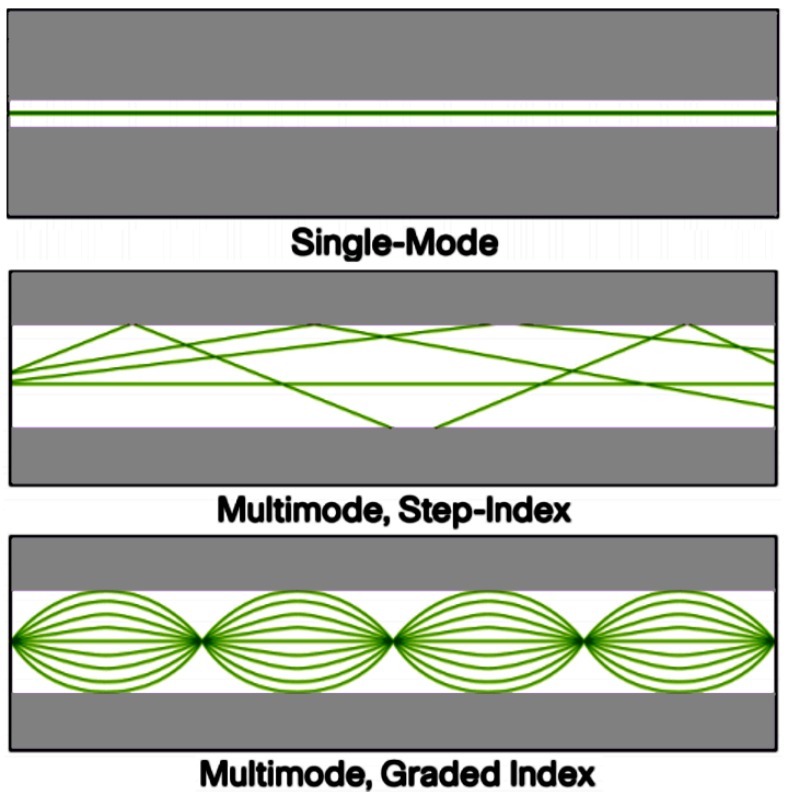
Multimode and single-mode fibers.

Single-mode fibers enjoy lower fiber attenuation than multimode fibers and retain better reliability of each light pulse because they have no dispersion associated with multiple mode fibers. Hence, data can be transferred over a longer distance. Similar to multimode fibers, the earlier single-mode fibers were commonly characterized as step-index fibers (shown in Figure 5), which means the refractive index of the fiber cladding is a step below that of the core rather than graduated as in the case of graded-index fibers. Current single-mode fibers have grown into a more sophisticated design like depressed clad, matched clad, or other mysterious structures.

**Figure 6:**
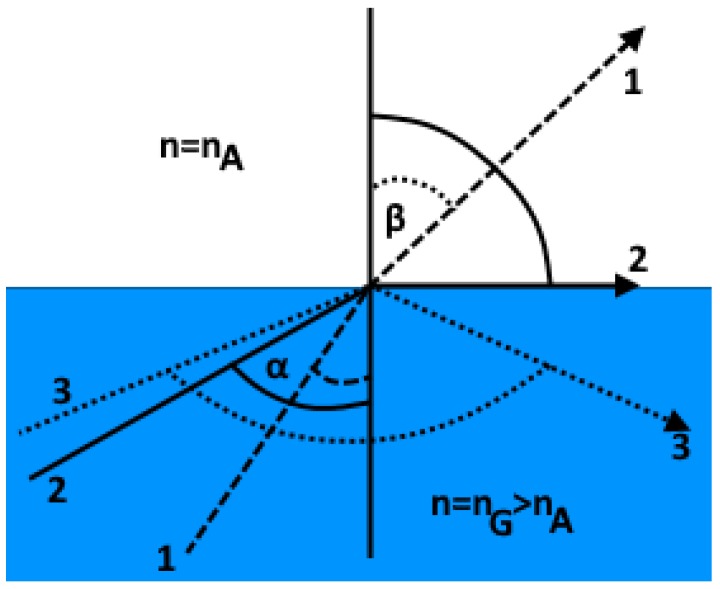
Total internal reflection phenomena

The core size of single-mode fibers usually is nine microns. Because only one mode can propagate down the fiber length, the total internal reflection process does not occur; hence, the concept of numerical aperture becomes similar to those of multimode fibers. The numerical aperture of multimode fibers is usually larger than those of single-mode fibers. The most common lasers appropriate for applications over single-mode fiber include distributed feedback (DFB) and Fabry–Perot lasers. The attenuation of single-mode fibers is about 0.2 dB per km [43]. Optical fibers operate based on the principle of total internal reflection. Imagine rays of light striking a distinct boundary separating an optically less dense medium. A less dense medium is the one with a lower reflection index. At an appropriate incidence angle, these rays rather than passing through will be reflected fully. This phenomenon is referred to as the total internal reflection [44,45]. Prisms in binoculars and camera viewfinders make use of total internal reflection. If the incidence angle is represented by the symbol (*α*), and the angle of refraction as *β* (see Figure 6 at this boundary to the less-dense medium, (*n_G_* > *n_A_*) assuming air and glass are being considered), the condition *α* < *β* holds. However, the angle *β* cannot be greater than 90°. This is evident considering the Snell’s law of refraction (Equation (1)):

(1) $$\frac{\sin\;\alpha }{\sin\;\beta }=\frac{n_{A}}{n_{G}}$$

Bearing in mind that (sin *β*) cannot be greater than one,

(2) $$\sin\;\alpha_{critical}=\frac{n_{A}}{n_{G}}<1$$

For even greater angles of incidence, the rays of light are entirely reflected back into the denser medium almost without encountering any loss. It is the same principle that guides light around bends as well as inside optical fibers [46].

Optical fibers have applications for assisting us in various aspects of our lives—for example, in amplifiers [47], in telecommunications [48,49], in medicine [50], in aerospace and aviation technology [51,52], in engineering [53,54], nanotechnology [55,56], and in sensing applications. Optical fiber sensors have been studied for over 40 years. Several concepts have been suggested, and many methods have been established for various parameters as well as for various uses. Commercialization of optical fiber sensors has been carried out successfully. However, out of the many methods investigated, only a small amount of applications and methodologies have been commercialized successfully [57]. The optical fiber-based sensors possess many advantages over copper cables for their high sensitivity, small size, large bandwidth, lightweight quality, as well as immunity towards electromagnetic interferences [58,59,60]. Pressure, temperature, and strain are the extensively investigated parameters, and, for the optical fiber sensors, the Bragg fiber grating sensors are the most widely studied technologies. However, in various applications, optical fiber-based sensors are expected to compete with other existing technologies like electronic-based systems. To get attention, since customers are already familiar with the current technologies, there is a need to demonstrate the superior qualities of optical fiber-based sensors over other contemporary methods. Usually, customers are not interested in the procedures involved in the detection. However, these clients only desire sensors with excellent performance at reasonable costs. Therefore, optical fiber-based sensors should be obtainable in the form of a system that includes signal detection and signal processing.

## 5. Classification of Optical Fiber Sensors

**Figure 7:**
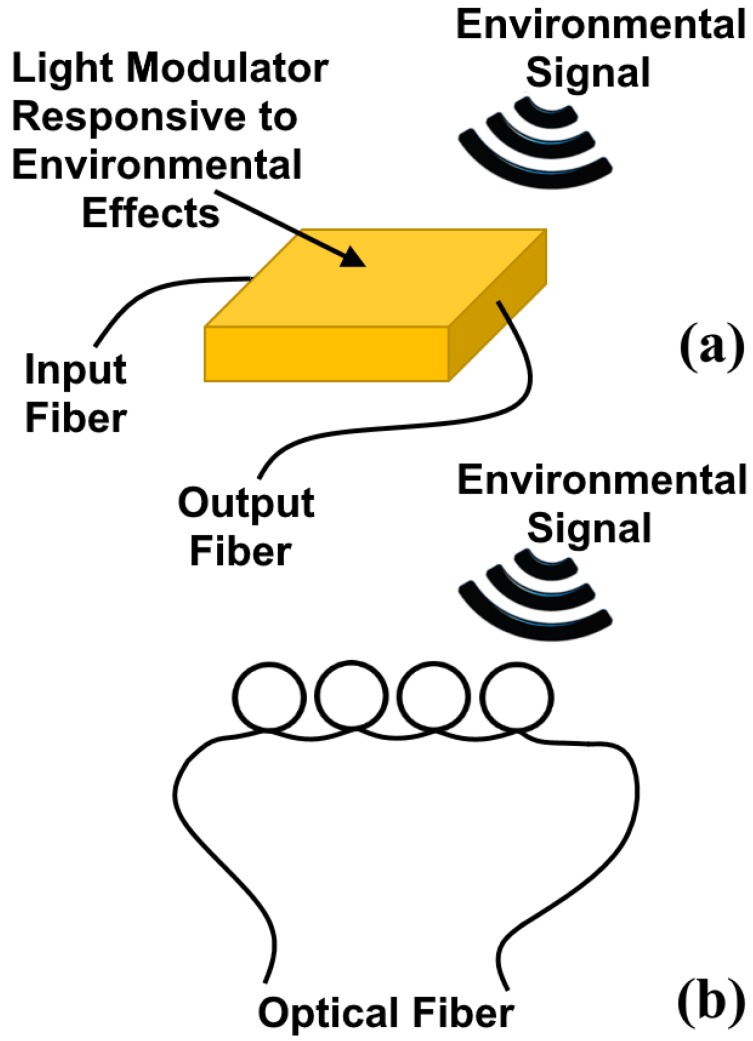
Schematic comparison between (**a**) extrinsic and (**b**) intrinsic sensors

There have been some approaches to the classification of optical fiber sensors. The increasing complexity of several types of optical fiber sensors is what prompted the development of adequate and appropriate classification systems. Factors such as physical quantity transduced by the sensors, detection systems, as well as sensor type have been considered in so many classifications. To develop the most suitable classification scheme for optical fiber sensors, an emphasis is given to the most important aspects and, hence, a classification method is adopted. Previous work that attempted to offer classification methods that cover the majority of the essential optical fiber sensors is cited in [61]. With the continuous increase in the development of optical fiber sensors, so many classification systems that were adopted previously became unsuitable. Other classification systems were given based on the modulation type chosen [62,63]. Hence, factors like wavelength, intensity, phase, and polarization were regarded as the primary classification standards. The disadvantage of this type of classification, however, is that the technique used is given emphasis rather than the sensor itself. This may be insignificant in applications where the most suitable technology is targeted for measuring a parameter of interest like pressure or temperature. This second method that considered variables like temperature, pressure, magnetic field, electric field vibration and flows in classifying sensors has also been adopted [64]. However, this approach is also associated with some disadvantages when applied in a similar way to the other methods of measuring various parameters like displacement. Other factors such as novelty and geographical location were also considered in the classification of sensors [65]. In the most extensively used system of classification, optical fiber sensors are classified as intrinsic or extrinsic sensors [66,67]. Extrinsic sensors are those in which the fiber guides the light wave, and the interaction between the magnitude of the parameter measured and light occurs outside the fiber. These types of sensors have been used successfully for some applications. For intrinsic sensors, on the other hand, interactions between light and the measured parameter occur inside the fiber. Figure 7 shows a comparison between the intrinsic and the extrinsic optical fiber sensors [61].

An important parameter to be considered in intrinsic sensors is the nature of the optical guidance of the fiber—that is, whether it is multimode, single, or otherwise. Another important sub-class of the intrinsic sensors is interferometric sensors [61].

### 5.1. Intrinsic Optical Fiber Sensors

Optical fibers can be applied as sensors in measuring temperature, strain, pressure or other parameters through fiber modification in such a way that the parameter of interest controls the polarization, intensity, wavelength, phase, and the time in light passes through the cord. The simplest sensors are those that vary the light intensity because they require only a simple detector and source of light. Intrinsic sensors can offer distributed detection for comprehensive coverage. This broad sensing ability associated with intrinsic sensors is very useful [68]. An optical fiber that has a temporary loss, which depends on temperature, can be used to measure temperature. This measurement can be possible by analyzing the Raman scattering of the optical fiber. Nonlinear optical effects that can change the light polarization, which depends on electric field or voltage, can be used in sensing electrical voltage. Other types of fibers are specially designed for special applications such as direction recognition [69,70,71]. Other optical fibers have applications in sonar and seismic detection. Examples of these types of fibers are hydrophones. Oil industries, as well as the navy in some countries, make use of the hydrophones systems. Microphone systems that involve the use of optical fibers have been developed by Sennheiser (Germany). In applications where high electric or magnetic fields are required, optical fiber based headphones and microphones are very useful. These applications include team communication among medics working on a patient in an MRI (Magnetic resonance imaging) system during surgeries that are MRI-guided [72]. In oil industries, optical fibers are used to measure temperature and pressure in oil wells [73,74]. These types of applications very much require optical fiber sensors since they can withstand very high temperatures compared to the semiconductor sensors. Optical fiber sensors can be used for interferometric sensings such as fiber optic gyroscopes, which are utilized for navigation in some cars and the Boeing 767 aircraft (USA). Optical fibers are used in making hydrogen sensors. Some optical fiber sensors have been designed for simultaneous measurement of collocated temperature and strain with high precision using Fiber Bragg gratings [75]. This approach is predominantly beneficial when obtaining data related to complex or small configurations [76]. Sensors based on Fiber Bragg grating are also very suitable for remote sensing. Detection of temperature and strain over considerable distances of up to 120 kilometers is also possible using “Brillouin scattering effects” [77]. 

Fiber-optic sensors have also found applications in electrical changeover gear for transmission of light between an electrical arc-flash to a digitally protecting relay in order to allow fast falling off a breaker to decrease the arc blast energy [78]. Fiber optic sensors that are based on Fiber Bragg grating improve performance, productivity, and protection in some manufacturing processes. Integration of Fiber Bragg grating technology enables sensors to offer full investigation and complete information on insights with precise resolution. These types of sensors are normally used in various industries such as aerospace, automotive, telecommunication, and energy. Fiber Bragg gratings are sensitive to mechanical tension, static pressure, and compression and changes in fiber temperatures. Central wavelength adjustment of light emitting source provides the effectiveness of Fiber Bragg grating optical fiber sensors [79,80]. The structure of the side-polished fibers (SPFs) has a cladding section that is partially removed on one side; therefore, by modification of the cladding, the evanescent field of the propagating light within the core can interact with surrounding materials that present different refractive indices. 

**Figure 8:**
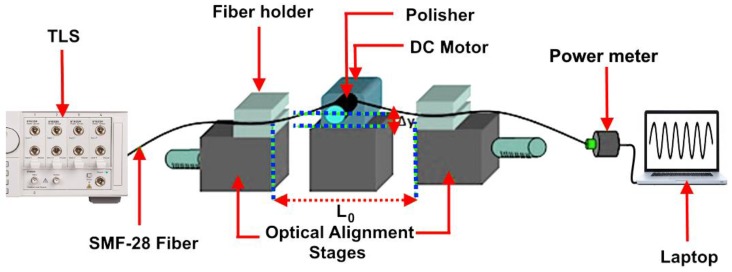
Polisher design setup.

Researchers have investigated many applications of the SPFs, especially in nonlinear optics photonics technologies [81,82]. We can illustrate the setup of the fiber modification as shown in Figure 3. In this case, a single mode fiber type that is known as SMF-28 is used for the fabrication, where it should be tightly suspended above the polishing wheel when the polishing process starts. The polishing section is only a few centimeters. Therefore, the SMF-28, which is striped, is suspended over the polishing wheel as illustrated in Figure 8. The SMF-28 should be adjusted in such a way that the center of the stripping section should be placed at L_0_/2.

**Figure 9:**
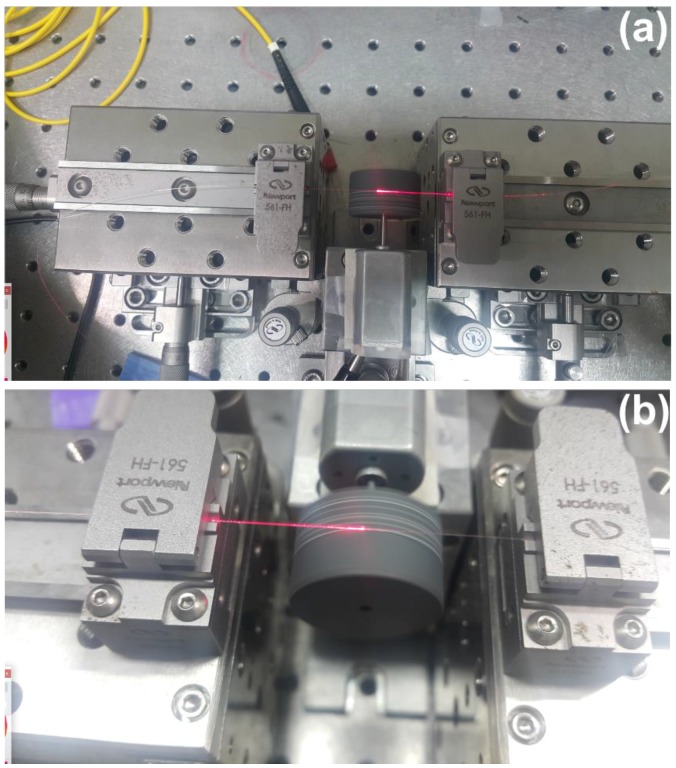
Polisher design setup; (**a**) the stage used to hold the fiber; and (**b**) the polishing process, where the light is figuring out from the fiber due to a removal of the cladding.

The double-sided scotch tape is wrapped around a shaft of the DC motor. Therefore, the silicon carbide paper sticks to the double-sided scotch tape to create the uniform polishing wheel in such a way that it is perpendicular to the suspended SMF-28 (Figure 9). The position of the polisher should be adjusted to create the contact between the fiber and the wheel. Figure 9 shows the experimental fabrication of the SPF. 

### 5.2. Extrinsic Optical Fiber Sensors

This type of fiber optic sensor makes use of optical fiber cables, usually the multimode type, to pass controlled light from either an electronic sensor linked to an optical transmitter or a non-fiber optical sensor. The advantage of extrinsic sensors is that they extend to places that cannot be otherwise accessible—for example, measuring the inside of aircraft engines using fibers to pass radiation to a radiation pyrometer that is situated on the exterior part of the machines. Similarly, extrinsic fiber optic sensors can be utilized in measuring the internal temperature of electrical transformers, in which the presence of a high electromagnetic field makes it impossible to measure using other measurement techniques. Extrinsic fiber optic sensors offer an outstanding shield of the frequency signal from being corrupted by noise. Regrettably, several traditional sensors release electrical outputs that must be changed to optical signals for fiber use. Extrinsic sensors found application in measuring temperature, rotation, acceleration, vibration, velocity, as well as displacement [83].

## 6. Fiber Bragg Grating and Applications

**Figure 10:**
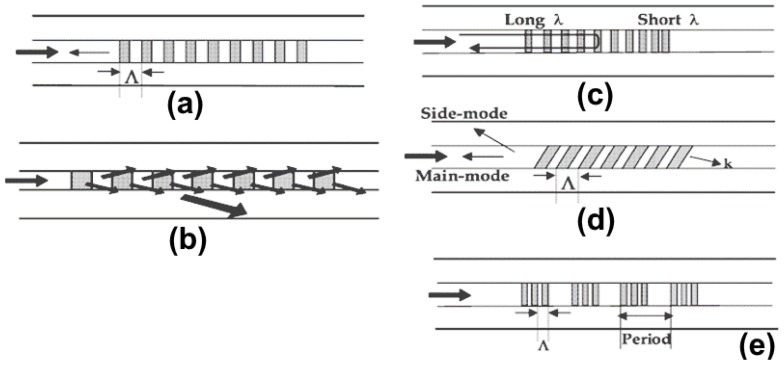
Types of fiber gratings. (**a**) Fiber Bragg grating; (**b**) long-period fiber grating; (**c**) chirped fiber grating; (**d**) tilted fiber grating; (**e**) sampled fiber grating

Even though the development of fiber gratings has been reported on since 1978 [84], it was only in 1989 that serious research on fiber gratings begun. This serious research activity followed the discovery of the regulated and operational methodology for their fabrication [85]. Fiber gratings have been widely used in amplifier gain flattening filters, fiber laser, and dispersion compensators for optical communication purposes. Rigorous studies have also been carried out on fiber grating sensors, and thus many have been commercialized. Different types of fiber gratings are shown in Figure 10.

**Figure 11:**
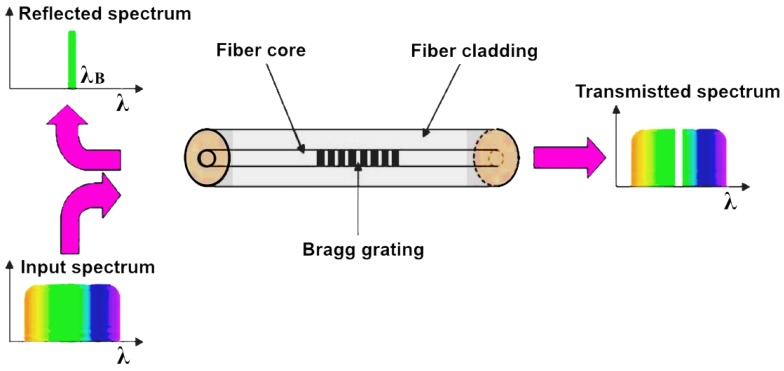
Schematic representation of the principle of Fiber Bragg grating.

A Fiber Bragg Grating (FBG) is a periodic perturbation of the refractive index alongside some meters on the fiber length. In the design of FBG, the core is exposed to ultraviolet light [86]. The perturbation index inside the single-mode fiber core serves as a filter that reflects incident optical fields. The reflection of the incident optical field is maximally achieved when the perturbation index and the wavelengths of the incident fields match by [57]:

*λ_B_* = 2*n_eff_A* (3)

where *λ* is the grating period and *n_eff_* is the effective index of refraction of the fiber (see Figure 11). If the grating period changes, or if the effective index of refraction changes, due to changes in temperature or applied strain, the grating period and the effective refractive index will also change, thereby shifting the wavelength of the mean reflectance. These characteristics can be utilized for the purpose of sensors [86].

There has been an increasing demand for sensors in almost all spheres of modern technology. The use of sensors that are based on Fiber Bragg grating technology has the potential to provide a lasting solution [87]. The strengths of distributed sensors can be further harnessed either through changing the sensitivity parameters of the FBG sensors or through coupling of both pressure and temperature sensors on one fiber. These types of sensing principles that are multi-parameter-based have been illustrated in [88,89]. Moreover, a remarkable range of operational temperature between 37 and 573 K has been demonstrated in [90]. The oil and gas industries processes in severe situations or space are potential marketplaces that nowadays assent to and value the sensors that are based on Fiber Bragg grating technologies. Individual markets try to use these technologies in diverse ways and most are very successful. An example of current advances in the oil and gas industries is Fiber Bragg gratings-based flowmeters that can be utilized in the downhole and harsh surface conditions, where temperatures can be above 573 K and have pressure of 99 atmospheres [89,90]. A substantial additional market where Fiber Bragg grating technologies have been widely recognized over the years is in structural health monitoring. In building constructions, bridges and many other types of large structures, Fiber Bragg grating sensors are employed to monitor continuously and verify the structural quality of these structures [91]. Optical fiber sensors like Fiber Bragg grating sensors are appropriate for composite material process monitoring because of their low invasiveness. The advantage of Fiber Bragg grating over other sensors is that they allow access to some physical parameters in the material. Hence, they can be used to examine the thick laminates and provide access to the manifestation of exothermic phenomena or residual strains [92].

## 7. Waveguides and Applications

**Figure 12:**
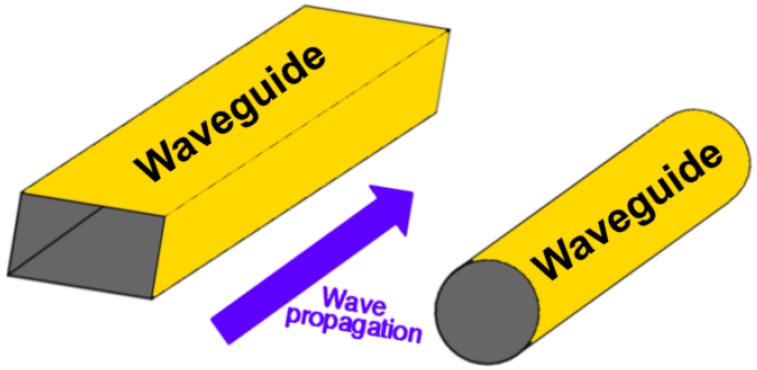
Rectangular and circular waveguides.

A waveguide consists of a hollow, metal tube that is a unique form of transmission line. The technology of applying hollow pipes to streamline the movement of electromagnetic waves first appeared in 1897. The 1930s, following the development of the first microwave-producing equipment, necessitated the creation of a hollow waveguide for them. The success of these hollow waveguides motivated scientists to invent waveguides in the infrared region of the electromagnetic spectrum. These waveguides were initially used for medical purposes, but other areas of applications followed (Figure 12). [93].

**Figure 13:**
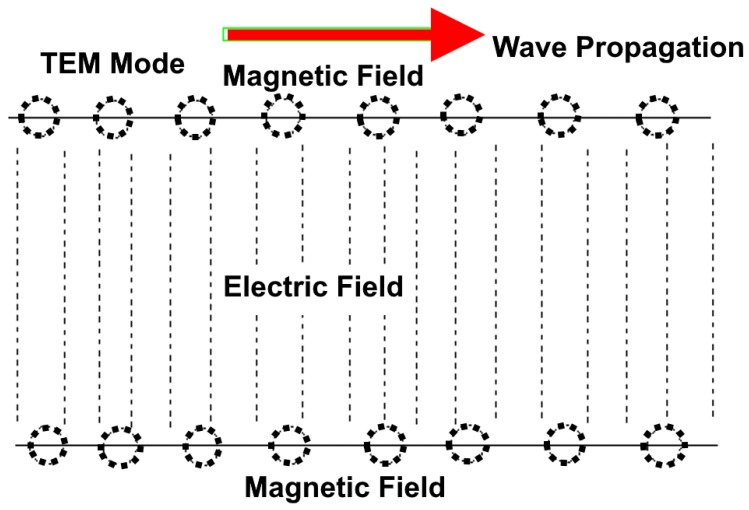
Transverse electromagnetic (TEM) mode propagation of a waveguide.

It directs the waves in a similar way river banks head a tidal wave [93]. Nevertheless, since waveguides are regarded as single-conductor materials, there is a difference in the way electrical energy is propagated down a waveguide as compared to the way in which it is propagated through a two-conductor transmission system. Figure 13 shows the propagation of the TEM mode in the waveguide. 

From Table 1, it could be concluded that a major improvement in waveguides is the decrease in propagation losses. The waveguide dimensions become impossibly large for lower frequencies, while, when the rate is higher, the dimensions become impracticably smaller. Waveguides present numerous advantages when compared to their optical fiber counterparts. Waveguides can transmit wavelengths above 20 μm. Their air core enables them to deliver high power lasers. Waveguides have relatively simple structures and they are cheap compared to the existing optical fibers. These qualities allow their use in applications where transmission of electromagnetic radiation requires a material of high mechanical, optical and thermal properties. Even though waveguides are associated with some setbacks such as losses upon bending as well as small numerical aperture, they appear to be the best option for use in sensors. Waveguides can be classified into two classes based on the principles on which they operate. These are attenuated total internal reflection and leaky-type waveguides. In attenuated total internal reflection-based waveguides, hollow core substances are present, which are enclosed by a wall that has a refractive index lower than that of the wavelength of the transmitted light. In the design of metallic waveguides, the inner wall is formed using a smooth metallic surface by depositing a metal film on the inner surface of plastic or a glass tube. Dielectric waveguides, on the other hand, are achieved by the formation of alternating high-low refractive index structure formed by the addition of multiple dielectric layers onto the metal surface. There has been a dramatic increase in the use of substrate integrated waveguides over the last decade due to their compact, low profile, and many other advantages over the conventional metallic waveguides. They resemble the conventional waveguides in their performances and can be made with printed circuit boards [94]. 

The need to explore waves at a millimeter scale for the subsequent generation of mobile communications has contributed significantly to the advancement of modern telecommunications components based on microwaves [95]. Substrate-integrated waveguides have found many potential applications outside the telecommunication industry. They are widely used in the automotive industry, as well as in biomedical devices for sensing applications. In terms of their dispersion and propagation characteristics, substrate integrated waveguides are similar to rectangular waveguides. 

**Figure 14:**
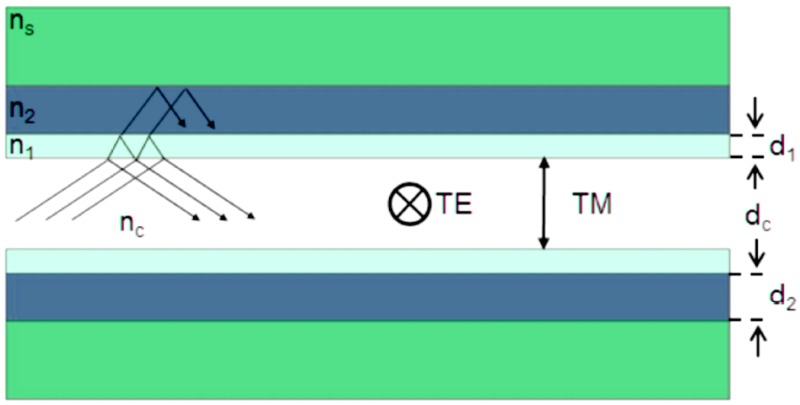
1D structure of narrow waveguide.

Another important emerging area is opted fluidics, which combines the benefits of optics and microfluidics in order to achieve highly compact and highly functional materials. Specifically, fluidic elements are integrated into the photonics structure [96,97]. A lot of optofluidic sensors are produced for healthcare and pharmaceutical researchers. Moreover, sensors are also available for biochemical analyses, environmental monitoring as well as biomedical researchers [98]. Integration of optical and fluidic structures can simply be achieved via optofluidic waveguides. Optofluidic waveguides are found to provide a very powerful foundation for building optofluidic sensors. The liquid core serves as the medium through which the light is guided with the help of highly reflective mirrors that are achieved by the sidewalls of the core. High sensitivity is usually achieved by taking advantage of flow or guidance of the light and the fluid through the same medium or channel. This direct interaction provides high sensitivity due to the small volume of the liquid as well as strong optical connections. Sensitivity can be achieved as low as the molecular level and this is the ultimate desire for any given analytical procedure. Just like other waveguide systems such as slot waveguides and photonic crystals, they have a high attractive capacity for optofluidic integrations with planar systems [99,100]. These types of waveguides allow simultaneous confinement and propagation of light because of the interference of light that occurs at the claddings that are made up of alternating thin layers of low- and high-refractive index. The operating principle of these types of waveguides can be understood easier when one considers the 1D structure as presented below in Figure 14. Assuming that two layers of the cladding are considered, and their refractive indexes are respectively *n*_1_ and *n*_2_ (with *n*_1_ > *n*_2_), propagation of light through the core is achieved by Fresnel reflections at the cladding interfaces. Repeated reflections at the interfaces cause the entrapped light to interfere. Interference cladding is designed to certify certain conditions to strengthen the intensity of the reflected light [101]. 

Presently, optical biosensors have emerged as the favorite choice to replace bulky laboratory instruments for such applications where a bulky quantity of samples is required to be simultaneously analyzed, like microplate array systems. Optical biosensors possess some desirable characteristics like small size, low-cost and being easier to use. These pleasant characteristics allow them to be used in online monitoring and sensing for the analysis of samples that are complex, and for real-time or online monitoring of experimental procedures [102]. Therefore, optical bio-sensing is now an active research field and commercialization of a number of platforms has already been realized. The application areas include environmental monitoring, clinical application, and food safety and control [103]. The most powerful and reliable biosensors are undoubtedly those based on fluorescence. Fluorescence intensity, decay time, and emission anisotropy are among the parameters that could be measured and used for sensing [104]. There is, therefore, a variety of options towards improving the performance of biosensors. Optical waveguides are dielectric configurations having two extreme wavelengths in the infrared and the ultraviolet regions of the electromagnetic spectrum and can be used to transport energy between these two extreme wavelengths. Based on their geometrical shape, they can be categorized into two main classes: planar and cylindrical. Optical waveguides are contained within the first group and are comprised of a cylindrical central dielectric core-cladding by an element characterized by a low refractive index (by z 1%). The planar waveguides are prepared from a dielectric slab core enclosed between two layers of the cladding with slightly lower refractive indices [105]. In both layers, propagation of light and streamlining along the direction of core depend on the famous total internal reflection phenomenon. When light is propagated within a planar or cylindrical waveguide, the light is reflected totally at the interface between two mediums in which one is optically denser provided that the angle of refraction is greater than the critical angle. In fluorescent biosensors that are based on evanescent field excitation, the geometry of the sensing region must be properly designed in order to optimize the excitation ability along the probe length, and also to prevent the coupling of the emitted fluorescence into guided modes that do not propagate in the cladding. This is known as “V-number mismatch” [106,107].

Many industrial operations such as welding and cutting are achieved using carbon dioxide lasers. However, bringing the laser beam from the source of flame to the desired area is a challenge because other equipment may block the path. The only method to successfully deliver the beam is using articulated arms; however, these colossal systems also require large spaces and mirrors requiring regular maintenance and alignment. Attempts have been made to use solid core waveguides to deliver high power carbon dioxide beams, but, due to thermal damage, particularly at the high interface, they have not been successful. Short lifespan associated with some of the promising solid core is also a disadvantage. Transmission of up to 3000 Watts of laser power is required at the initial attempt using circular and rectangular metal-coated waveguides. However, large core radii are needed to achieve the needed power levels and, when subjected to bends and other movements, these large radii waveguides exhibit poor outputs. Moreover, most of the industrial operations require low order outputs to achieve sharp and clean cuts. Therefore, to attain this type of quality with hollow waveguides, smaller size core is needed to filter and transmit the higher order mode in the desired fashion. It is a known fact that, as the size of the core decreases, the loss of the waveguide increases and this, in turn, decreases the power capacity. These setbacks reduce the use of hollow waveguides to industrial uses such as marking cutting of plastic or paper, which requires lower power [108].

**Figure 15:**
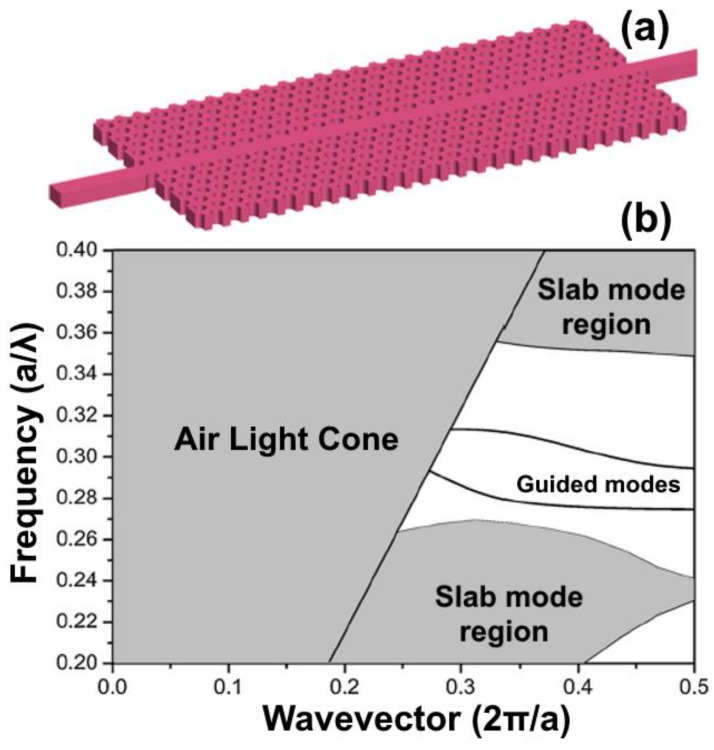
(**a**) Photonic crystal waveguide slab; (**b**) photonic band diagram

Controlling and streamlining the movement of light has been one of the main focus areas of research in the last few decades. Materials such as optical fibers, which operate based on the principle of total internal reflection, have significantly transformed the communication industry [109]. The concept of photonic crystals was proposed independently by John [110] and Yablonovitch [111] in 1987. They use the electronic band concept analogous to semiconductor crystals. Photonic crystals are dielectric materials that are periodic in 1D, 2D or 3D orthogonal directions. They can be fabricated in a simple way and have unusual optical properties. Two-dimensional photonic crystals are the most interesting and can be categorized into two: dielectric materials in air, or air in the dielectric material. The former is fabricated easily via periodic inscription of holes in materials of high dielectric properties such as GaAs, Si, and Ge. Photonic bandgaps are characterized by photonic crystals because of the intermittent disparity in the refractive index. Photonic band gaps have a range of frequencies that cannot allow propagating inside the crystal. Because of this peculiar property, waveguides are formed through inducing of line imperfections in photonic crystal structures [112,113]. These line faults are used to guide light from one place to another. The defects are guided inside the photonic band gap through the streamline via the total internal reflection principle. Because of the asymmetrical boundary, this streamlining creates backscattering, which causes slow light phenomena. Slow light causes optical signals to compress in space, and this enables interaction between light and matter and allows miniaturization. Presentation of photonic crystal waveguides is given in Figure 15a. A variety of dielectric slab materials of the high refractive index can be used to produce photonic crystal slabs. A typical example of these photonic crystals is based on polymethyl methacrylate (PMMA) prepared by [114]. A photonic crystal slab based on Si_3_N_4_ has been demonstrated to work in the visible region of the electromagnetic spectrum. Moreover, photonic crystals based on InP/InGaAsP structure have been prepared with a slight loss [115].

Several types of research are on-going for the potential applications of photonic crystals. Most frequent among them are related to the photonic integrated circuits. The introduction of defects can be achieved through the photonic band gap. Instead of guiding light through total internal reflection, it could be conducted using line defects in photonic crystals. The use of a photonic bandgap to guide light allows for small bending loss even when the bending angles are large. In the area of sensors, photonic crystals have been widely used in the field of sensors. A photonic crystal slab provides sensitivity to the photonic band gap. Some of these sensors can be designed to detect pressure using a GaAs/AIGaAs slab [116].

## 8. Conclusions

Waveguides and optical fibers have applications for assisting us in various aspects of our lives. As anticipated, optical fiber-based sensors can be appropriate instruments for monitoring physical parameters such as strain and temperature. A review of some of the recent advances related to the design and application of optical fiber sensors has been given. It has been established that optical fiber grating sensors and side-polished fibers continue to play a significant role in the development of various sensors with the combination of new fiber materials and structures. These new classes of Fiber Bragg grating sensors have the potential for many industrial uses. Each market and application has its separate advantages derived from Fiber Bragg grating based sensor applications. Nevertheless, optimization of the sensor systems is not restricted to the sensors only, but the entire system must be considered. To obtain an optimal Fiber Bragg grating sensors system for application, optimization of the interrogator with regard to wavelengths, resolutions, sweep frequencies as well as costs, among other factors, must be considered.

Fiber optics sensors have been developing for many years but have not achieved great commercial success yet due to the difficulties of introducing modern technologies that could replace current well-established technologies. However, for applications such as sensing in high-voltage and high-power machinery, or in microwave ovens, the fiber optics sensors are well recognized for presenting many advantages. Fiber Bragg grating sensors have developed significantly, and these can now be used to monitor conditions within the wings of airplanes, in wind turbines, bridges, large dams, oil wells and pipelines. In smart structures, which are the main drivers for the further development of fiber-optic sensors, the fiber sensors can monitor and obtain essential information about the strain, vibrations, and other phenomena. Since the year 2000, fiber optics has provided a significant contribution in applications such as optical communications, transmission fibers used underwater, in terrestrial areas, metro and local area networks (LAN). Other special fibers have been used in amplifiers, lasers, sensors and photonics devices. Further improvements of the fiber optics can be done by providing higher bandwidth, transmissions capacities for longer distances, and introducing devices with at a lower cost. For instance, in the LAN fiber world, the use of new wideband multimode fibers is recommended to improve the overall system efficiency. The wideband multimode fibers can be used in wider frequency ranges from visible to infrared such as the short wavelength-division multiplexing ranges 850 to 950 nm. Another rapidly growing technology is free-space communication, where the optical signals can be used for satellite–satellite communications. Recently, optical fibers have been used for transmission from light emitting sources such as high-power lasers, where the sudden changes in wavelength can be controlled easily in these devices. 

## Figures and Tables

**Table 1:** Rectangular waveguide sizes (source: https://www.everythingrf.com/tech-resources/waveguides-sizes).

Waveguide Name			Recommended Frequency (GHz)	Cutoff Frequency Lowest Order Mode (GHz)	Cutoff Frequency Next Mode (GHz)	Inner Dimensions of Waveguide Opening	
EIA	RCSC	IEC				A Inch	B Inch
-	WG9	-	2.20 to 3.30	1.686	3.372	3.5	1.75
WR340	WG9A	R26	2.20 to 3.30	1.736	3.471	3.4	1.7
WR284	WG10	R32	2.60 to 3.95	2.078	4.156	2.84	1.34
-	WG11	-	3.30 to 4.90	2.488	4.976	2.372	1.122
WR229	WG11A	R40	3.30 to 4.90	2.577	5.154	2.29	1.145
WR187	WG12	R48	3.95 to 5.85	3.153	6.305	1.872	0.872
WR159	WG13	R58	4.90 to 7.05	3.712	7.423	1.59	0.795
WR137	WG14	R70	5.85 to 8.20	4.301	8.603	1.372	0.622
WR112	WG15	R84	7.05 to 10	5.26	10.52	1.122	0.497
WR102	-	-	7.00 to 11	5.786	11.571	1.02	0.51
WR90	WG16	R100	8.20 to 12.40	6.557	13.114	0.9	0.4
WR75	WG17	R120	10.00 to 15	7.869	15.737	0.75	0.375
WR62	WG18	R140	12.40 to 18	9.488	18.976	0.622	0.311
WR51	WG19	R180	15.00 to 22	11.572	23.143	0.51	0.255
WR42	WG20	R220	18.00 to 26.50	14.051	28.102	0.42	0.17
WR34	WG21	R260	22.00 to 33	17.357	34.715	0.34	0.17
WR28	WG22	R320	26.50 to 40	21.077	42.154	0.28	0.14
WR22	WG23	R400	33.00 to 50	26.346	52.692	0.224	0.112
WR19	WG24	R500	40.00 to 60	31.391	62.782	0.188	0.094
WR15	WG25	R620	50.00 to 75	39.875	79.75	0.148	0.074
WR12	WG26	R740	60 to 90	48.373	96.746	0.122	0.061
WR10	WG27	R900	75 to 110	59.015	118.03	0.1	0.05
WR8	WG28	R1200	90 to 140	73.768	147.536	0.08	0.04
WR6	WG29	R1400	110 170	90.791	181.583	0.065	0.0325
WR7	WG29	R1400	110 to 170	90.791	181.583	0.065	0.0325
WR5	WG30	R1800	140 to 220	115.714	231.429	0.051	0.0255
WR4	WG32	R2200	172 to 260	137.243	274.485	0.043	0.0215
WR3	WG32	R2600	220 to 330	173.571	347.143	0.034	0.017
